# Stable isotopes reveal the effect of trawl fisheries on the diet of commercially exploited species

**DOI:** 10.1038/s41598-017-06379-6

**Published:** 2017-07-24

**Authors:** Hilmar Hinz, Joan Moranta, Stephen Balestrini, Marija Sciberras, Julia R. Pantin, James Monnington, Alex Zalewski, Michel J. Kaiser, Mattias Sköld, Patrik Jonsson, Francois Bastardie, Jan Geert Hiddink

**Affiliations:** 1 Mediterranean Institute for Advanced Studies (UIB-CSIC), C/ Miquel Marquès, 21, 07190 Esporles, Balearic Islands, Spain; 2 Instituto Español de Oceanografía, Centro Oceanográfico de Baleares, Moll de Ponent s/n, 07015 Palma de Mallorca, Spain; 3 School of Ocean Sciences, Bangor University, Menai Bridge, Anglesey, LL59 5AB United Kingdom; 4 Department of Aquatic Resources, Institute of Marine Research, Swedish University of Agricultural Sciences, Turistgatan 5, SE-453 30 Lysekil, Sweden; 5 Technical University of Denmark, Institute for Aquatic Resources, Section for Ecosystem based Marine Management, Charlottenlund Castle, DK-2920 Charlottenlund, Denmark

## Abstract

Bottom trawling can change food availability for benthivorous demersal species by (i) changing benthic prey composition through physical seabed impacts and (ii) by removing overall benthic consumer biomass increasing the net availability of benthic prey for remaining individuals. Thus trawling may both negatively and positively influence the quantity and quality of food available. Using *δ* ^13^C and *δ* ^15^N we investigated potential diet changes of three commercially exploited species across trawling gradients in the Kattegat (plaice, dab and Norway lobster (*Nephrops*)) and the Irish Sea (*Nephrops*). In the Kattegat, trawling affected primarily the biomass of benthic consumers, lowering competition. *Nephrops* showed significant positive relationships for *δ* ^13^C and a domed relationship for *δ* ^15^N with trawling. In the Irish Sea, intense trawling had a negative effect on benthic prey. *δ* ^13^C and *δ* ^15^N thus showed the inverse relationships to those observed in the Kattegat. Plaice from the Kattegat, showed a significant relationship with trawling intensity for *δ* ^13^C, but not for *δ* ^15^N. No relationship was found for dab. Changes of *δ* ^13^C and *δ* ^15^N correlated with changes in condition of species. The results show that the removal of demersal competitors and benthos by trawling can change the diets of commercial species, ultimately affecting their body condition.

## Introduction

Demersal fisheries are widespread on continental shelves^1^ and are known to cause significant physical changes to benthic ecosystems through the use of heavy ground ropes, chains and otter boards dragged across the seabed that drive fish into nets^2^. Bottom trawl fisheries affect populations of commercial species, such as flatfish and crustaceans, directly by harvesting, or indirectly through physical habitat changes induced by mechanical trawl equipment leading to population changes in benthic invertebrates on which these benthivorous species feed^3–5^. So far most studies on bottom trawling have examined these direct and indirect effects separately. The consequences of the simultaneous change in the population density of commercially exploited species and their benthic food resource as a result of bottom trawling have largely remained understudied^3^.

The harvest of conspecifics or species with a similar diet by the fishery may directly reduce competition for food in systems that are prey-limited^3^. Conversely, reduced prey biomass and changes in prey compositions through the mortality caused by fishing gear impacts on benthos may reduce the quantity and quality of prey available to predators^5–9^. Therefore, whereas the release of consumers from competition for prey may result in higher food availability and higher body condition, alternatively the reduction in prey availability may result in lower energy intake and lower body condition. Fishing, thus, has two opposing effects on individuals of commercially exploited species and depending on which of these is more important, the consequences of bottom trawling on food intake of the individual may be either positive or negative. It is therefore the ratio of consumer to prey biomass that becomes important in determining whether prey availability has relatively increased or decreased in response to trawling.

Furthermore bottom trawling may alter prey composition and availability for scavenging benthic consumers by increasing the accessibility to food in the short-term, as benthic fauna are left damaged or dead in trawl tracks after the passage of trawl gear^10, 11^ or alternatively by unwanted catches being discarded back into the sea^10, 12, 13^. Many commercial fish species including flatfish and gadoids, are known to display scavenging feeding behaviour^14^ and it can be assumed that most demersal species can act as scavengers. However, if these temporary increases in food availability translate into measurable long term benefits for scavenging species is thus far unknown.

Changes in fish diet have been generally assessed through the study of stomach samples^15^. However, stomach contents analysis presents a number of methodological biases, i.e. the abundance of prey organisms in stomachs will be biased towards those species that are digested more slowly, and it only provides an estimation of what the fish have ingested in the hours prior to capture. Stable isotope analysis of muscle tissue can be used to detect changes in the diet of species over longer times scales^15, 16^ and is particularly useful for species such as crustaceans that macerate their prey, making identification of prey taxa in stomachs extremely difficult. The isotopes *δ* ^13^C and *δ* ^15^N are widely used for this type of analysis. *δ* ^15^N values increase from prey to consumers considering the two taxonomic groups studied (crustaceans and fish) by approximatively 2.5‰ to 3.4‰ per trophic transfer with each trophic step^17–19^ and thus *δ* ^15^N value is used to indicate the trophic position of a species^20, 21^. *δ* ^13^C, in contrast, increases only by ~1‰ with each trophic step^22^. As a consequence of the conservative transfer properties of *δ* ^13^C, it has been used to trace carbon sources in systems where differences in *δ* ^13^C values between different primary producers (i.e. the carbon source) are distinct^23, 24^.

The current study builds on from studies by Hiddink *et al*.^6, 25^ and Johnson *et al*.^5^ on *Nephrops* fisheries in the Kattegat and Irish Sea, which demonstrated that changes in benthic prey availability due to trawling appears to be a key driver for plaice (*Pleuronectes platessa*) and Norway lobster (*Nephrops norvegicus*) condition but not for dab (*Limanda limanda*). The present study aims to increase the insights gained by these previous studies by investigating the qualitative changes in commercial species diets (plaice, dab and *Nephrops*) reflected by changes in isotopic signatures along trawling gradients in both the Kattegat and in the Irish Sea. The two study areas covered distinct parts of the fishing effort continuum. Trawling intensities in the Kattegat were lower ranging between 0.2 to 7.8 times trawled per annum (y^−1^) (the total area swept by trawl gear within a given area of seabed, divided by that seabed area), due to various spatial effort regulations i.e. a No Take Zone and seasonal closures, while in the Irish Sea trawling intensities were higher with intensities ranging from 2.7 to 11.9. y^−1^. In particular, we predicted for *Nephrops*, the species primarily exploited by the fisheries, that the response in isotopic signatures would be distinct for the two study areas. We hypothesized that in the Kattegat where trawling affected the biomass of benthic consumers, consequently lowering competition, diets would be composed of higher level trophic prey leading to increases in both *δ* ^13^C and *δ* ^15^N. In contrast, we predicted that in the Irish Sea, where the high intensities of trawling had a negative effect on benthic prey biomass, that the trophic level of the diet would decrease reflected by lower *δ* ^13^C and *δ* ^15^N values. These suggested relationships followed the assumption that benthic prey spectra for *Nephrops* may have been simplified by intense benthic feeding by competitors and fishing impacts. Moreover, we predicted that condition would increase with increases in both *δ* ^13^C and *δ* ^15^N values, as higher trophic level benthic prey tends to be nutritionally more valuable compared to lower trophic level prey^26^. Furthermore, we investigated the response of isotopic signatures of plaice and dab to trawling in the Kattegat and the link of these with stomach content and condition.

## Results

**Figure 1:**
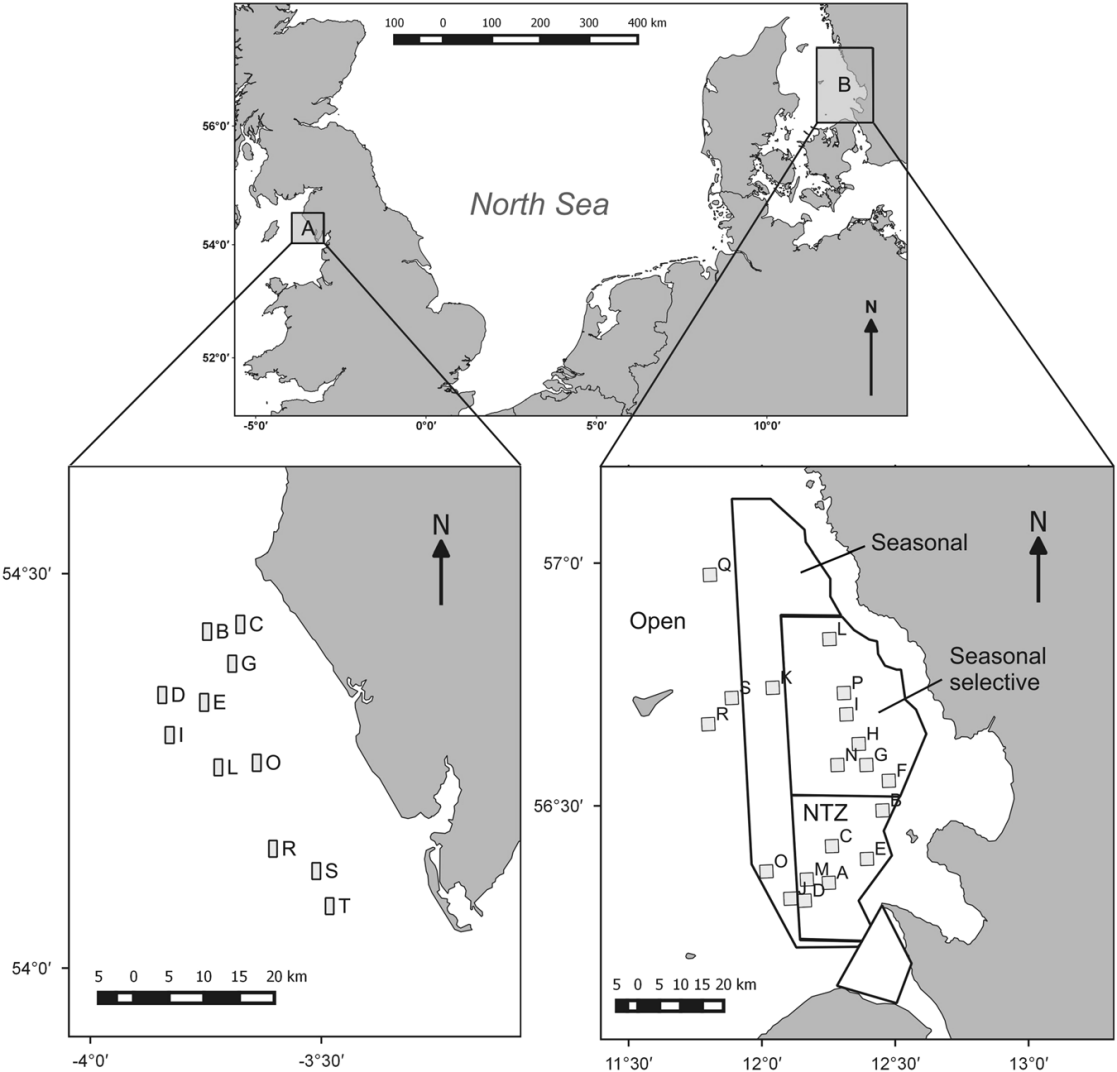
Location of the two *Nephrops* trawling areas with sampling stations imposed (**A**) north eastern Irish Sea and (**B**) Kattegat. In the Kattegat fishing effort management zones are displayed, NTZ = No Take Zone. Maps were created with the software package QGIS Lyon V2.12.

**Figure 2:**
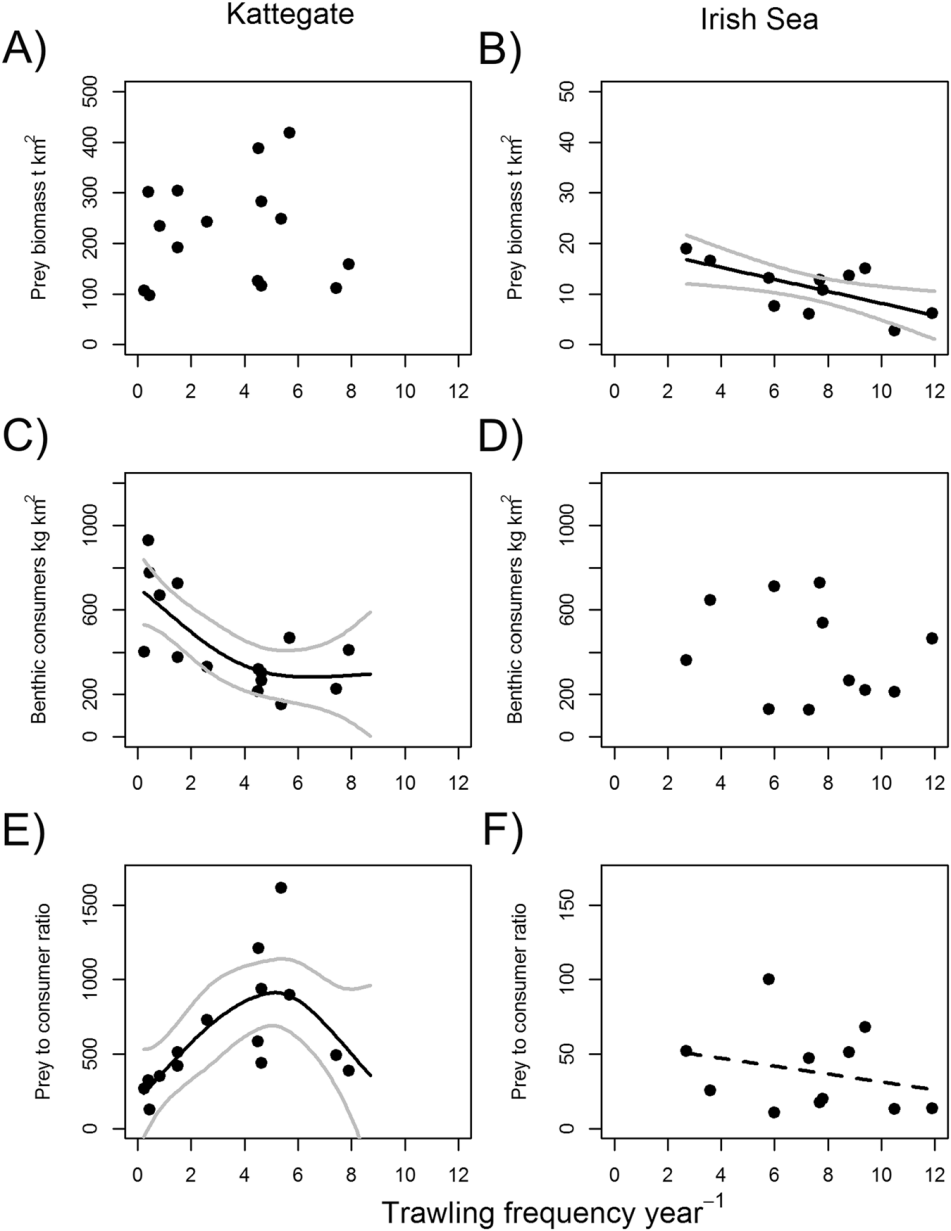
(**A,B**) Response of benthic biomass to trawling frequency (**A**: Kattegat, **B**: Irish Sea). (**C,D**) Response of main benthic consumers (Plaice, Dab and *Nephrops* combined and additionally long rough dab in the Kattegat) to fishing pressure (**C**: Kattegat, **D**: Irish Sea. **E,F**). Note that y-axis scales of panels (**A**, **B**) as well as (**E**,**F**) are different. Benthic prey biomass to benthic consumer ratio (kg km^2^) in relation to fishing intensity (**E**: Kattegat, **F**: Irish Sea). Black lines represent significant GAM model relationships and grey lines their 95% confidence intervals. Dashed line in **F** signifies non-significant trend. Points represent mean values at stations.

Comparing benthic prey and consumer biomasses in the Irish Sea and the Kattegat sampling areas (Fig. 1) demonstrated that trawling affected these two components differently in respective locations. Benthic prey biomass (excluding large infauna, see Methods) in the Irish Sea showed a significant negative relationship with increasing trawling intensity, while in the Kattegat no significant relationship was detected (Fig. 2A,B, Table 1). Benthic consumer biomass significantly declined with increased trawling in the Kattegat, but not in the Irish Sea (Fig. 2C,D, Table 1). In the Kattegat there was a significant dome shaped relationship between the benthic invertebrate biomass to consumer ratio with a maximum at fishing intensities between 5–6 times trawled per annum. In the Irish Sea benthic prey biomass to consumer ratio was not related to trawling intensity (Fig. 2E,F, Table 1).

**Table 1:** GAM and GAMM statistical outputs for response variables tested individually against the independent variable trawling frequency y^−1^ (results also displayed in Fig. 2A–F).

	N	Res.df	F	R^2^	p	
**Kattegat**						
Benthic prey biomass (kg km^−2^)	15	13.18	4.312	0.005	0.666	
Benthic consumers (kg km^−2^)	15	12.76	6.863	0.512	0.008	**
Benthic prey: consumer ratio (kg km^−2^)	15	12.23	4.982	0.474	0.023	*
**Irish Sea**						
Benthic prey biomass (kg km^−2^)	11	10.00	6.78	0.366	0.028	*
Benthic consumers (kg km^−2^)	11	10.00	0.47	0.057	0.509	
Benthic prey: consumer ratio (kg km^−2^)	11	10.00	0.65	0.036	0.442	
*Nephrops* condition (g)	199	195.00	6.42	0.931	0.097	

Res.df are the residual degrees of freedom.

**Figure 3:**
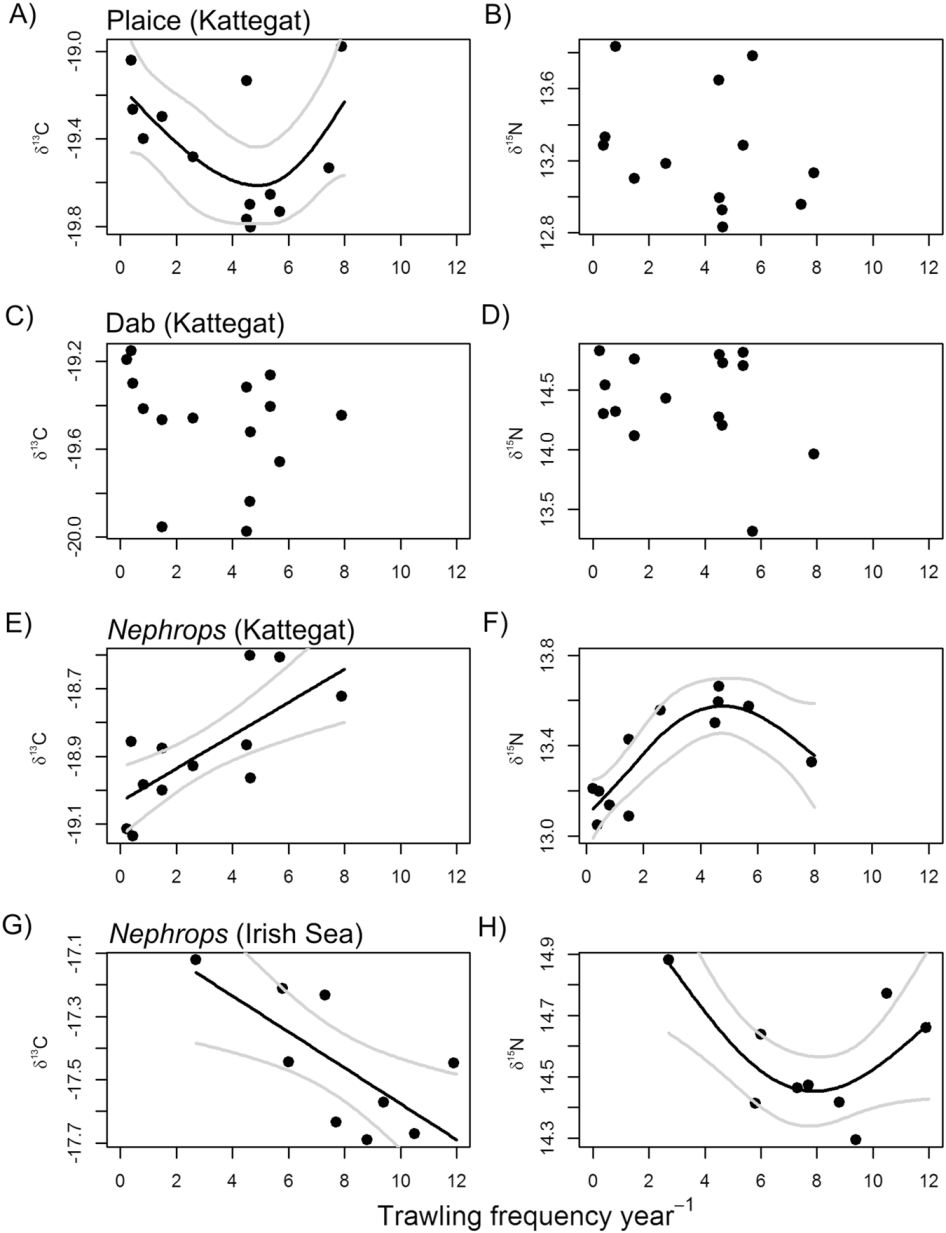
Relationship of isotopic carbon and nitrogen signatures along trawling gradients in muscle tissue of (**A,B**) Plaice in the Kattegat, (**C,D**) Dab in the Kattegat (**E,F**) *Nephrops* in the Kattegat and (**G,H**) *Nephrops* in the Irish Sea. Solid black lines signify significant GAMMs and grey lines represent 95% confidence intervals. Points represent mean values at stations.

The GAMM analysis of *δ* ^13^C and *δ* ^15^N revealed significant responses with increasing trawling intensity for both *δ* ^13^C and *δ* ^15^N for *Nephrops*, but only for *δ* ^13^C for plaice from the Kattegat (Fig. 3 and Table 2). For plaice, *δ* ^13^C was lowest at trawling intensities of 4 to 6 times trawled per annum, but higher at low and high trawling intensities (Fig. 3A). *Nephrops* showed significant, but opposite, relationships with trawling for both *δ* ^13^C and *δ* ^15^N in the Kattegat and in the Irish Sea (Fig. 3E–H). Whereas *Nephrops δ* ^13^C increased linearly with trawling intensity in the Kattegat, *Nephrops δ* ^13^C decreased with trawling in the Irish Sea. In the Kattegat the relationship of *δ* ^15^N with trawling was domed with the maximum *δ* ^15^N values at trawling intensities around 5 times trawled per annum (Fig. 3F, Table 2). In the Irish Sea, a negative domed relationship was evident with minimal *δ* ^15^N values at intermediate trawling intensity between 6 to 10 times y^−1^ (Fig. 3H,). Dab did not show any significant relationship for *δ* ^13^C and *δ* ^15^N (Table 2).

**Table 2:** GAMM statistical outputs presented in Fig. 3 A-H.

	N	Groups	Res.df	F	R^2^	p	
**Kattegat**							
Plaice *δ* ^13^C	76	13	73.71	3.16	0.193	0.032	*
Plaice *δ* ^15^N	76	13	75.00	0.95	0.002	0.331	
Dab *δ* ^13^C	85	15	83.13	2.05	0.083	0.212	
Dab *δ* ^15^N	85	15	84.00	1.68	0.043	0.199	
*Nephrops δ* ^13^C	68	12	67.00	12.17	0.219	<0.001	***
*Nephrops δ* ^15^N	68	12	65.45	9.68	0.331	<0.001	***
**Irish Sea**							
*Nephrops δ* ^13^C	52	9	8.00	7.615	0.172	0.008	**
*Nephrops δ* ^15^N	52	9	7.12	5.730	0.157	0.021	*

Relationship of the response variables listed with trawling frequency y^−1^. Res.df are the residual degrees of freedom.

To ensure that isotopic baselines variations did not drive or influence the relationships observed between isotopic values and trawling, predicted isotopic baselines from empirical models, using bottom temperature as an environmental predictor, were correlated with the mean isotopic values observed over the sampling stations using least square regression models. No correlations were evident between the predicted baselines and observed mean isotopic values (S3). Furthermore, the GAMM analyses examining the relationship between isotopic signatures and trawling were repeated on the observed data subtracting predicted isotopic baseline data (i.e. the new response variable was the difference between observe isotopic values – the predicted baseline). GAMMs showed almost identical trends as well as significance levels as observed in the original analysis (see S4 and S5), demonstrating that predicted baseline variation within sampling areas had significant influence on the relationships reported due to trawling.

**Figure 4:**
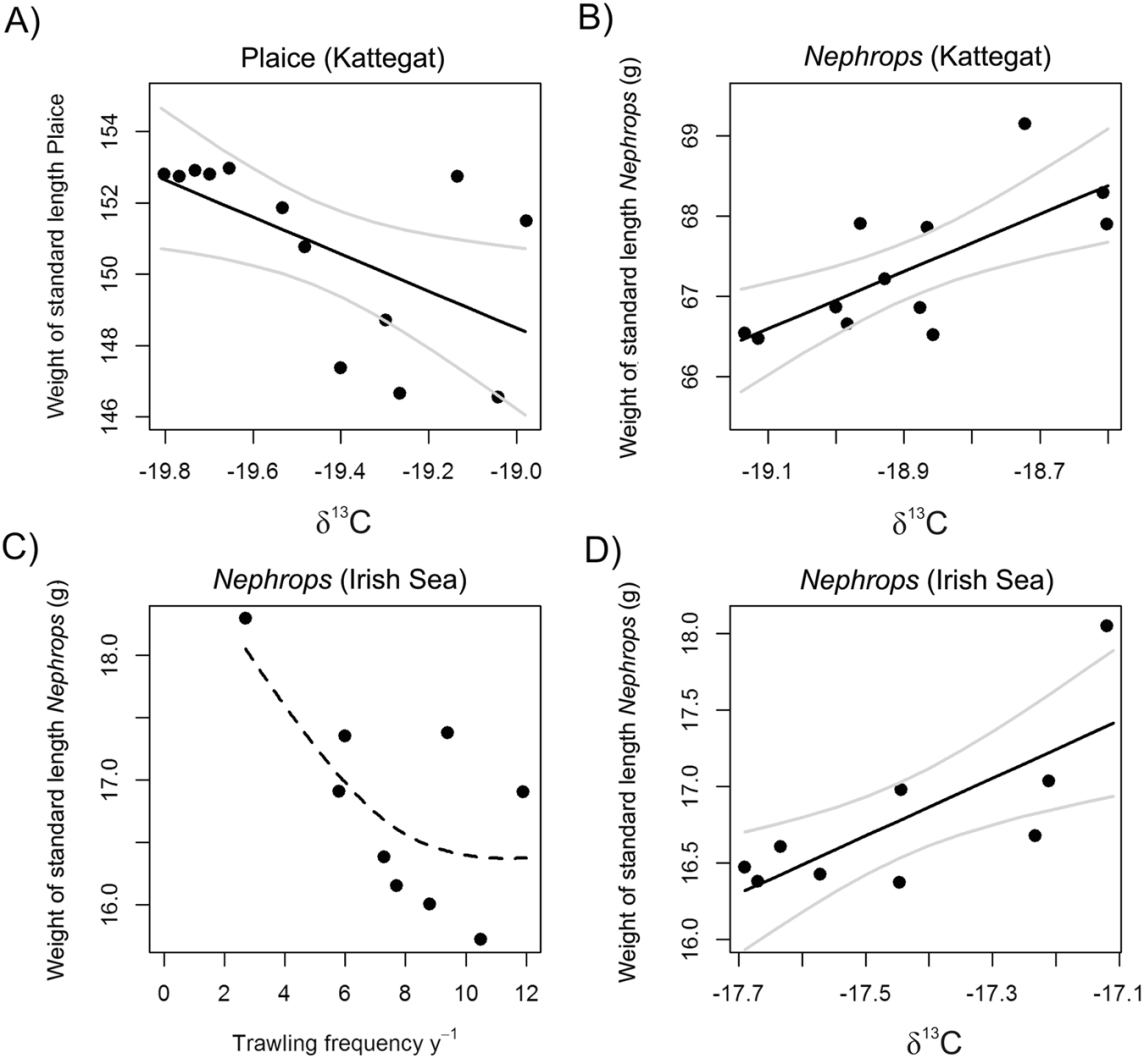
Relationship between *δ* ^13^C and condition as weight of a standard length fish (**A**) plaice and (**B**) Nephrops in the Kattegat. (**C**) Weight of standardized length Nephrops (g) with increasing trawling frequency in the Irish Sea. Standardized length for *Nephrops* refers to carapace length in all instances. Only significant relationships are presented except for (**D**), the relationship between *δ* ^13^C and condition expressed as weight of a standard length *Nephrops* from the Irish Sea. The dashed line represents the non-significant prediction of the GAMM model. *Nephrops* weight displayed in the Irish Sea are predictions for clawless male individuals, see Methods. The significant relationship of δ^15^N and *Nephrops* condition in the Kattegat (Table 3) was not displayed due to lower r^2^ and significance values compared to δ^13^C.

The GAMM for *Nephrops* condition (expressed as the weight of a standardized length individual) over the trawling gradient in the Irish Sea was marginally non-significant (Fig. 4C, Table 1). Due to the closeness to significance and the contextual indications from the Kattegat data, GAMM condition predictions were used to investigate the relationship between *δ* ^13^C and *δ* ^15^N values and condition. The relationships between trawling intensities and body condition for *Nephrops*, dab and plaice in the Kattegat were not presented within the present publication as they formed part of a previous publication^25^. Summarizing these results, a significant negative linear trend of *Nephrops* condition was observed with increasing trawling intensity in the Kattegat, while for plaice a domed relationship with its peak at approximately 5 times trawled per annum was detected. Dab did not show a significant response to increases in trawling intensities.

The relationship between isotopic signatures and predicted species condition were investigated using least square regression analysis of *δ* ^13^C (untreated and lipid normalized) and *δ* ^15^N versus species condition (previously published data^25^ for the Kattegat and the present publication for *Nephrops* for the Irish Sea see above) revealed a significant negative relationship for plaice with *δ* ^13^C. *Nephrops* condition in the Kattegat and the Irish Sea showed significant positive relationships with *δ* ^13^C (Fig. 4A,B,D, Table 3). No relationship between condition and *δ* ^13^C was found for dab. As the relationships detected between *δ* ^13^C and condition showed similar significance levels after lipid normalization it was concluded that the source of *δ* ^13^C changes observed were related to changes in prey species composition rather than related to the lipid content of the tissue. *δ* ^15^N showed a significant relationship with *Nephrops* condition in the Kattegat but not in the Irish Sea (Table 3, not displayed in Figure). No relationship was found between condition and δ^15^N for plaice or dab.

**Table 3:** Least square regression models of δ^13^C and δ^15^N against standardized body weight (as a proxy for condition).

		N	Res.df	F	R^2^	p	
**Kattegat**							
Plaice	*δ* ^13^C	13	11	5.49	0.33	0.038	*
	*δ* ^13^C _*normalized*_	13	11	7.27	0.39	0.021	*
*Nephrops*	*δ* ^13^C	12	11	10.93	0.52	0.007	**
	*δ* ^13^C _*normalized*_	12	11	8.022	0.44	0.017	*
	*δ* ^15^N	12	11	5.92	0.37	0.035	*
**Irish Sea**							
*Nephrops*	*δ* ^13^C	9	7	9.23	0.57	0.018	*
	*δ* ^13^C _*normalized*_	9	7	7.87	0.52	0.026	*
	*δ* ^15^N	9	7	2.52	0.26	0.156	

Weight values were obtained from the predicted GAMM model outputs and δ^13^C and δ^15^N observed measurements. Both δ^13^C and δ^13^C normalized were used in the analysis. A significant relationship of both δ^13^C and δ^13^C normalized verified that the relationship was not related to the lipid content of the tissue. Res.df are the residual degrees of freedom.

**Figure 5:**
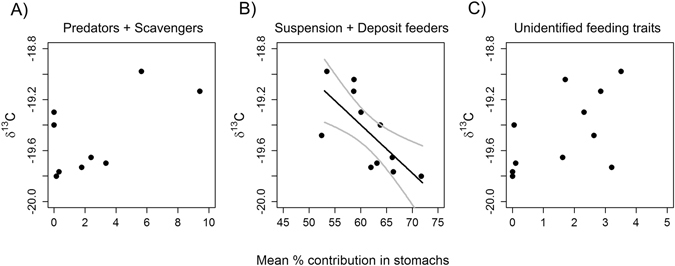
Plaice stomach composition of functional feeding groups along the trawling gradient in the Kattegat. Relationship between mean percentage contribution in weight in plaice stomach of (**A**) Predators and Scavengers, (**B**) Suspension + Deposit feeders, and (**C**) Unidentified feeding traits and *δ* ^13^C.

Stomach content compositions of plaice were analysed to explain the effects of trawling on *δ* ^13^C by the feeding type of the prey consumed. The mean percentage contribution of prey items in stomachs belonging to different feeding groups a) scavenging and predatory prey, b) suspension and deposit feeding prey, and c) non-determined feeding types were correlated with the measured *δ* ^13^C signature of plaice from the stations sampled. The percentage contribution of suspension and deposit feeding prey showed a significant negative relationship with *δ* ^13^C values (d.f. = 1,9, p = 0.014, r^2^ = 0.51), while no such relationship was found for the other groups (Fig. 5).

## Discussion

Towed bottom fishing gear have been shown to affect commercially exploited fish condition through two main drivers; changes in density of intra- and inter-specific competitors and changes in benthic prey biomass^5, 25^. Both processes are thought to directly affect both the quantity and quality of food intake of commercial species. The results of the carbon *δ* ^13^C and nitrogen *δ* ^15^N analysis demonstrated that trawling was most likely responsible for dietary changes in plaice and *Nephrops*, but not in dab. In the Kattegat, which due to spatial effort regulations had lower fishing effort ranges, trawling reduced primarily the overall numbers of benthic consumers while not significantly affecting benthic prey biomass. Thus, at low to medium trawling intensities, as trawling increased up to approximately 5 times trawled per annum, more benthic prey became available per benthic consumer. At higher trawling intensities, the trend reversed with less prey being available per individual. Conversely, in the Irish Sea fishing intensities were higher, leading to decreases in benthic prey biomass while not significantly affecting the numbers of benthic consumers. This absence of a significant response may have to do with demersal fish (in particular plaice and dab) immigrating into the relatively small area of the fishing ground from other areas or it may be related to consumer populations having already stabilized at low levels across the trawling gradient. Prey biomass in the Irish Sea was overall significantly lower compared to the Kattegat. Consequently, the prey to predator ratio was also lower emphasizing that overall competition for food may have been more intense in the Irish Sea.

The lower *δ* ^13^C of *Nephrops* at low trawling intensities in the Kattegat and at high trawling intensities in the Irish Sea could be the result of less optimal feeding conditions due to stronger competition for food resources. Two alternative changes in feeding and diet exist that could explain the observed patterns. *Nephrops* are known to adopt suspension-feeding or ‘micro-raptorial’ feeding during periods of food scarcity^27^. Thus, the lower *δ* ^13^C in the tissues of *Nephrops* could be explained by an increase in this feeding activity and the consumption of planktonic particulate organic matter that in general has lower *δ* ^13^C compared to benthic invertebrates^28^. Alternatively, *Nephrops* may also feed increasingly on supra-benthic mysids if abundances of other benthic prey are low i.e. due to competition or fishing impacts. Mysids have a stronger trophic association to the planktonic food and thus also have lower *δ* ^13^C^28^. However, which of the two mechanisms caused the observed patterns could not be determined by the presented data. δ^15^N followed the tendencies of δ^13^C only in part. In the Kattegat, an increase in δ^15^N was observed up to a trawling intensity of approx. 5 times trawled y^−1^ after which it slightly decreased. In the Irish Sea, δ^15^N decreased together with δ^13^C until a trawling intensity of approximately 8 times y^−1^ after which it slightly increased again. The reasons for these patterns is currently not clear but may have to do with a change in the dominant benthic prey consumed. *δ* ^13^C as well as *δ* ^15^N were positively correlated with the condition of *Nephrops* thus emphasizing the positive effect the switch from planktonic food web prey towards benthic prey had for the species.

Of the two flatfish species in the Kattegat, only plaice showed a significant relationship between *δ* ^13^C and trawling frequency. Plaice displayed the lowest values of *δ* ^13^C at trawling frequencies around 5 times trawled y^−1^. The decrease in *δ* ^13^C at medium trawling intensities was most likely associated to a change in the diet of plaice towards prey items that are more closely linked to carbon sources originating from planktonic food webs such as suspension and deposit feeding species^28^. Furthermore, *δ* ^13^C values of plaice showed a significant relationship with condition inferring that the suggested change in prey composition at medium fishing intensity reflected a change towards better feeding conditions. Feeding may be optimal for the individual at this point due to the presence of fewer competitors and relatively high densities of benthic prey. As this relationship was evident in both untreated and lipid normalized data the possibility that the negative relationship was caused by a higher lipid content of the fish tissue could be excluded. This result highlighted that increases in condition are not necessarily linked to diet changes towards higher trophic level prey as originally hypothesized, but that other dietary changes can equally affect fish condition positively. The absence of a response in isotopic signatures in dab appears to suggest that dab, with its generalist feeding strategy^5, 29^, is able to feed successfully despite competition and changes in prey availability without measurable effects on its condition.

Another source of dietary change that may influence the stable isotope signatures of commercial species may be through discards by fishery. Discarding of by-catch from the *Nephrops* fisheries is generally high and thus could be a potential source of food for the commercial species^30^. Dab, plaice and undersized *Nephrops* are likely to be the main discard species in both study areas^6, 25^. Based on our observation on fish biomass multiplied by the fishing frequency, discarding of these species should have steadily increased with trawling frequency in both the Kattegat and in the Irish Sea. Therefore, if discards formed an important part of the diet of *Nephrops* an increasing relationship of both *δ* ^13^C and *δ* ^15^N with trawling frequency should also have been evident in both areas, for increased feeding on fish and crustacean discards would have led to higher isotopic signatures compared to feeding on other benthic prey. While the relationships observed in the Kattegat matched this trend, in the Irish Sea *δ* ^13^C and *δ* ^15^N showed decreasing trends, opposite to the theoretical increases predicted. In contrast to *Nephrops*, plaice *δ* ^13^C and *δ* ^15^N values in the Kattegat did not show relationships that could be associated with discarding. It is therefore concluded that while discarding cannot be completely excluded as an explanation for the observed trends in *δ* ^13^C and *δ* ^15^N for *Nephrops* in the Kattegat, the data presented from the Irish Sea appears to point more towards shifts in feeding from benthic food towards a more planktonic based food source.

Few studies to date exist that examine the responses of commercially exploited species diet and condition through trawl induced changes in prey availability^3, 6^. Assessments of changes in stable isotope signatures of commercial species are even less common and currently few other studies exist that investigate changes in *δ* ^13^C and *δ* ^15^N along a trawling gradient. A study in the Celtic Sea investigated changes in *δ* ^15^N of four commercial fish species at low, medium and high trawling intensities^31^. This study found that whiting (*Merlangius merlangus*) at different size classes had significantly higher δ^15^N at low trawling effort sites, whilst a similar trend was only evident for large megrim (*Lepidorhombus whiffiagonis*). δ^15^N values for plaice and lemon sole (*Microstomus kitt*) showed no significant differences. δ^13^C was not analysed. The lower δ^15^N was interpreted as a change in diet of whiting and large megrim to a benthic diet at high trawling intensity sites. This was interpreted as reflecting scavenging of these fish on benthic organisms that were damaged by trawling activities. The relationships observed by the Celtic Sea study^31^ were quite different to the results of the present study. However, it needs to be noted that within the present study we examined the effect of trawling on the diet of relatively small flatfish and a crustacean species that are true benthivores, while in the Celtic Sea study significant relationships were found for whiting and large megrim, species that generally tend to have piscivorous diets. Studies in the Mediterranean Sea, investigating differences in isotopic signatures of δ^13^C and δ^15^N in trawled versus un-trawled areas found higher δ^15^N values in untrawled areas for red mullet (*Mullus barbartus*), hake (*Merlcuccius merluccius*), monkfish (*Lophius budegassa*) and pandora (*Pagellus erythinus*). No trawling related differences were found for δ^13^C^32–34^. The authors of these studies concluded that trawling only had limited trophodynamic effects on fish species. The lack of a clear response in δ^13^C and δ^15^N of commercial species in studies using trawled versus untrawled area comparisons may have to be reinterpreted in the light of results from the present study. When comparing trawled versus untrawled areas only two points along the trawling spectrum are being compared. Within our results we demonstrate that at very low and high trawling intensity, isotopic compositions may be quite similar as prey availability may be driven by two different processes, i.e. competition amongst benthic consumers and the reduction of benthic prey biomass through fishing impacts.

The changes in *δ* ^13^C and *δ* ^15^N documented by the present study were subtle and it could be argued that they simply reflect geographical differences in isotopic baseline values, therefore confounding results. However, we found no evidence of predicated isotopic baselines to correlate with mean observed values. Furthermore, repeating the GAMM analyses on the relationships between isotopic signatures and trawling using as a response variable the difference between observed values and predicted isotopic baselines showed almost identical trends and significance levels. These results suggested that baseline variations are a highly unlikely source for the observed trends. This argument was further supported by the distinct responses observed for plaice and *Nephrops* in the Kattegat. If baseline changes were at the root of the observed changes responses should have been similar. This, together with the correlation of *δ* ^13^C with specific prey types found in stomachs of plaice and correlations of body condition for plaice and *Nephrops* (for both areas) appears supportive of results reflecting genuine diet changes.

The findings of this paper highlight that trawling does not simply remove fish and benthos, but it also changes prey and predator relationships. As such it emphasises that the secondary effects of trawling should be taken into account within an ecosystem approach to fisheries. Fisheries management measures, such as the reduction or displacement of fishing effort trough fishing closures, may therefore have unexpected effects on both benthic prey and the predators^3^. These consequences need to be carefully evaluated and adapted to the local case scenarios to ensure the effectiveness of fisheries management measures. Based on the results of this study trawling frequencies of about 5 times y^−1^ may improve feeding and condition for plaice and *Nephrops* in the Kattegat. In the Irish Sea trawling should be no higher than 4 times y^−1^ to avoid negative effects on *Nephrops* diet and thus condition. Furthermore, by switching to gears that affects benthic prey to a lesser extent, such as gill nets, long-lines or pots and creels targeting *Nephrops* ^35^, the negative effects of bottom trawling on the diet and condition of commercial species may be mitigated.

## Materials and Methods

### Sampling areas

Survey work was conducted off the Cumbrian coast (UK) in May 2011 (north-eastern Irish Sea) and in the southern Kattegat in August 2013 (Fig. 1). Both areas are characterized by low-energy hydrodynamic conditions and have a muddy substratum and an active *Nephrops* fishery utilizing otter trawls^25, 36^.

### Fishing effort and station selection

Fishing effort in the Irish Sea was estimated from the spatial distribution of trawling activities over the fishing ground derived by combining over-flight data from 1999 to 2004, as in ref. 36, with Satellite Vessel Monitoring System (VMS) data from 2004 to 2008, as in ref. 6. For a more detailed description of fishing effort estimates see ref. 6. The 2008 fishing estimates were used within this study with the assumption that overall fishing patterns had not changed significantly over the following two years prior to the survey.

In the Kattegat, fishing pressure was estimated by combining VMS data with official logbook data and fishing gear dimension estimates. From this data, vessel tracks with an estimated impact area were calculated to estimate trawling frequencies over the trawling ground. For the calculation, only Swedish and Danish VMS and logbook data were used for vessels >15 m following the assumption that the fishing effort by other countries and vessels below 15m would be small and comparable to the effort distribution calculated. Data for a three-and-a-half-year period prior to sampling, from January 2010 to August 2013, was used to estimate trawling intensity (for more details see ref. 25).

In the Irish Sea, eleven sampling stations over a steep trawling gradient were selected for the sampling of fish and benthos. Trawling frequency ranged between 2.7 to 11.9 y^−1^. All stations had a similar depth (31 ± 6m) and substratum type (sandy mud) see Table S1.

The selection of stations in the Kattegat followed the same principles as in the Irish Sea. In total fifteen stations were sampled that had a trawling frequency gradient of 0.2 to 7.8 y^−1^. All stations had similar habitat characteristics with respect to depth (32 ± 3m) and sediment type (sandy mud) see Table S2.

Multivariate statistical analyses were used in both areas to ensure comparability of environmental variables of sampling stations included in the analysis. The results of these analyses have been published for the Irish Sea^36^ and for the Kattegat^25^.

### Study species

Tissue samples of plaice and dab for stable isotope analysis were collected from the Kattegat Sea, whereas tissue samples of *Nephrops* were collected from both the Kattegat and the Irish Sea. All three species are caught by commercial trawl fisheries, and are benthic predators feeding on relatively similar prey items. Plaice generally feed on small polychaetes and to a lesser extent on bivalves^5, 29, 37^, while dab feed on mobile crustaceans, polychaetes, fish, mollusc siphons and (the arms of) brittle stars^11, 38, 39^. *Nephrops* feeds on a great diversity of prey, in particular small crustaceans, molluscs, echinoderms (including brittle stars) and fish^40, 41^. Furthermore, it has been suggested that *Nephrops* may be able to adopt filter-feeding or ‘micro-raptorial’ (feeding on plankton or particulate organic matter) feeding during periods of food scarcity^27^, which is thought to be particularly important for females spending prolonged periods in their burrows during egg bearing.

### Sampling of benthos and fish

The benthic infauna was sampled by taking five 0.1 m^2^ grab samples at random locations within a station. In the Irish Sea, a station was defined by a 1 × 2 km box and in the Kattegat by a 3 × 3 km box. Each grab sample was sorted over a 1 mm sieve and preserved in 4% formalin for later taxonomic identification. Results from the five individual grabs were pooled before statistical analyses was carried out as within station variability was not of interest to this study. Only individuals with heads, in the case of worms, and oral discs, in the case of brittle stars, were included in the abundance estimates, whilst all parts where used for biomass estimates.

In the Irish Sea, fish were sampled during daylight hours using a rock hopper otter trawl with a net opening of 16 m, a head line height of 3 m, and an 82 mm diamond mesh cod-end. Two 30-minute tows were executed at 4 stations at a speed of 3 knots. Due to adverse weather conditions all remaining stations were only towed once. Similarly, in the Kattegat, fish were sampled using an otter trawl with a net opening of 25 m and an 80 mm cod-end. Two 30-minute tows were conducted at all stations. All fish from each haul were identified, measured to the nearest millimetre and weighed to the nearest gram on-board the vessel. If the number of fish caught per species was greater than 50 individuals, a subsample of 50 individuals was measured and weighed. Total abundance and weight of fish caught were measured per tow and standardized by the swept area to kg km^−2^. In the Kattegat, the carapace length (CL) and total body weight of *Nephrops* was recorded. In the Irish Sea, *Nephrops* catches were frozen directly after capture and taken to the laboratory for later analysis. During defrosting of bulk samples in the laboratory animals frequently had missing claws. As a consequence, all front claws (chelipeds) were removed prior to weighing.

Procedures of animal sampling were carried out according to relevant guidelines and regulations. Sampling protocols were approved by Bangor University.

### Sampling for fish stomach contents

Stomach samples of plaice and dab were taken from individuals with a body length range between 182–299 mm and 168–274 mm respectively in the Kattegat. Concentrating on these sizes limited the possibility of introducing ontogenetic changes in diet. Stomachs of 20 individuals per station were extracted and stored in 8% formalin solution.

### Sampling and stable isotope analysis

White muscle tissue samples from 5–6 individuals of each respective study species were collected for each station. For flatfish, individuals were chosen from the same size range as the stomach samples to evade confounding ontogenetic variation in carbon *δ* ^13^C and nitrogen *δ* ^15^N readings^28^. (Dab mean length: 198 mm ± 17 S.D.; Plaice mean length: 251 mm ± 29 S.D.). The mean carapace length (CL) for *Nephrops* collected in the Kattegat was 51 mm ± 1 S.D. and was 43 cm ± 3 in the Irish Sea. For the flatfish, a 2 × 2 cm piece of white muscle tissue was taken with the skin removed approximately 2 cm to the right of the pectoral fin and 1 cm above the lateral line. For the *Nephrops*, white muscle tissue was extracted from the first tail segment with shell, digestive and nerve tract removed. Due to logistical constraints of the sampling, all samples from the Kattegat were preserved in 100% pure ethanol for transport to the laboratory. Ethanol preservation of tissue samples has been shown to affect stable isotope readings in particular for carbon *δ* ^13^C and less so for nitrogen *δ* ^15^N^42^. Thus care needs to be taken when interpreting the absolute carbon *δ* ^13^C values. However, as all samples from the same area were treated the same, the relative values amongst stations with different fishing histories remain comparable. *Nephrops* samples from the Irish Sea were stored frozen at −18 °C after capture for later analysis in the laboratory. In the laboratory, all tissue samples were oven dried for 24 hours at 75 °C. Each individual tissue sample was ground to a homogenous powder using an agate pestle and mortar. Subsequently, 1.5–2.0 mg of the resulting powder was weighed into tin capsules for stable isotope analysis. Tissue samples were combusted for C and N isotope compositions using continuous flow isotope ratio mass spectrometry (CF-IRMS) THERMO delta X PLUS mass spectrometer (Scientific Technical Services - University of the Balearic Islands). The isotope mass spectrometer was operating in dual mode measuring *δ* ^15^N and *δ* ^13^C in the same sample. Stable isotope ratios where expressed in the conventional *δ* notion as parts per-mil (‰) according to the following equation 1:

1 $$\delta X=[({R}_{{\rm{sample}}}/{R}_{{\rm{standard}}})-1]\times 1000$$

where *X* is *δ* ^13^C or *δ* ^15^N and R is the corresponding *δ* ^12^C or *δ* ^15^N ratio respectively. The analytical precision of measurements expressed as the standard deviation was 0.16‰ for *δ* ^13^C and 0.11‰ for *δ* ^15^N as estimated from the bovine liver standard (1577b, US Department of Commerce, National Institute of Standards and Technology, Gaitherburg, MD) analysed together with the samples.

### Statistical analysis

To demonstrate how the effect of trawling on benthic prey and consumers differed in the two areas the effect of trawling frequency (times trawled y^−1^) on benthos (biomass), benthic consumers, and benthic consumers to benthic invertebrate prey biomass ratios were analysed using Generalized Additive Models (GAMs) for both areas. GAMs were used as the relationships were not expected to necessarily follow a linear relationship^25^. The response variable ‘biomass’ was total benthic infaunal biomass sampled per station, excluding the biomass of rare large infauna species such as large echinoderms and bivalve species (e.g. *Asterias rubens, Echinocardium spp*. or *Arctica islandica*) as they are not sampled well using grabs and are not eaten by our study species but disproportionately affect sample biomass. The main benthic consumers in the Irish Sea were plaice, dab and *Nephrops* (97% of biomass in otter trawls^6^). In the Kattegat these same three species and additionally long rough dab (*Hippoglossoides platessoides*) were the four main benthic consumers (89% of biomass in otter trawls^25^).

The main focus of the present study was to determine the effect of trawling on the stable isotope signatures of plaice, dab and *Nephrops*. As the relationships were not expected to necessarily follow a linear relationship the response of *δ* ^13^C or *δ* ^15^N with trawling intensity was modelled using Generalized Additive Mixed Models (GAMM). GAMMs were run using the package *mgcv* in R^43, 44^. A mixed model approach, with stations as a grouping variable, was used as the measurements of individuals caught at the same station were not independent. All models had a Gaussian error distribution. Homogeneity of residuals was established for each model through visual examination of plotted standardized residuals versus fitted values.

Isotopic baseline values of *δ* ^13^C or *δ* ^15^N may vary in space due to differences in environmental conditions, in particular temperature^45–47^. In general, to determine the isotopic baselines studies tend to collect tissue samples of primary consumers such as filter feeding bivalves or other basal organisms^45–47^. Organisms that were suitable for estimating isotopic baseline values, such as clams, were present in grab samples but no single species had a high enough biomass across all stations over the trawling gradient to allow estimation of isotopic baseline values. We therefore estimated the isotopic baseline using empirical relationships that relate the baseline to environmental conditions^45–47^. Here, we used the relationships that predict isotopic baselines from queen scallops tissue, *Aequipecten opercularis*, based on the annual mean sea bottom temperature (SBT) that was developed for the North Sea [δ^13^C_scallop_ = 0.336*SBT-21.53 (from^45^ modified by^47^); *δ* ^15^N_scallop_ = 0.59*SBT + 1.62 from^46^]. Mean annual bottom temperature data for the two-year period prior to sampling was used. Bottom temperature data was extracted from ocean models that can be accessed through the COPERNICUS MARINE ENVIRONMENT MONITORING SERVICE. (http://www.marine.copernicus.eu/; Irish Sea model data: NORTHWESTSHELF_REANALYSIS _BIO _004 _011; Kattegat model data: BALTICSEA _REANALYSIS _PHY _003 _008). To ensure that isotopic baselines variations did not drive or influence the relationships observed between isotopic values and trawling; predicted isotopic baselines were correlated with the mean isotopic values observed at sampling stations for respective species and areas. The robustness of our results were further examined by repeating the GAMM analyses using the difference between observed isotopic values and the predicted baseline and examining if it affected observed relationships. A persistence of trends and significance levels would indicate that the predicted isotopic baselines were unlikely be the source of the observed trends.

Similarly, the impact of fishing on the condition of the three commercial species was analysed by using Generalized Additive Mixed Models (GAMMs) to model the relationship between log_10_ weight and log_10_ length and trawling intensity, with station as a grouping factor and a Gaussian error distribution as described^6^. Thereafter, we refer to changes in the condition in the weight at length as the condition. For presentation purposes, condition is reported as the weight at a standardized length, which is the mean length observed considering the data from all stations. As the interaction between log_10_ length and trawling was not significant, this term was excluded. Homogeneity of residuals was assessed visually as described above. *Nephrops* condition was only calculated using male individuals.

The relationship between isotopic signatures and predicted species condition were investigated using least square regressions. To determine if any observed relationships between condition and *δ* ^13^C were caused by the lipid content of the tissue rather than by changes in prey composition, regression of *δ* ^13^C were performed with untreated and lipid normalized data following the arithmetic correction for aquatic animals^48^. Fish and shellfish in general do not store lipids in white muscle tissue^15^, nevertheless a study on lobsters^49^ found that lobsters that fed on the same food but with different lipid concentrations showed differences in *δ* ^13^C. Higher lipid content food had lower *δ* ^13^C. Thus, changes in *δ* ^13^C in white muscle tissue of study species with trawling intensity may either reflect that prey were linked to a different carbon source or indicate changes in the lipid content of the tissue. To distinguish between the effects of lipids in lowering *δ* ^13^C values and genuine changes in prey, we predicted that any observed relationship between *δ* ^13^C and commercial species condition should disappear after arithmetic lipid correction of *δ* ^13^C if higher lipid storage in the tissue was the responsible driver

2 $${\delta }^{13}{C}_{normalized}\,={\delta }^{13}{C}_{untreated}-3.32+0.99\,\times C:\,N$$

The resulting normalized *δ* ^13^C (equation 2) is comparable to the *δ* ^13^C after direct chemical extraction of lipids^48^.

Stomach content composition of plaice were analysed to explain the effects of trawling on *δ* ^13^C (see results section). *δ* ^13^C signatures were thought to be most likely influenced by the feeding type of the prey consumed. It was assumed that an increase in the amount of suspension feeders and deposit feeders compared to predators and scavengers in the diet may lead to a decrease in *δ* ^13^C as these feeding types are more closely related to the pelagic food web having themselves lower *δ* ^13^C signatures^50^. Thus, the relationship between δ^13^C and the mean percentage of prey feeding types using wet weight as an estimate of biomass in stomachs were examined by least square regressions. Prior to analysis the prey items were categorized into three feeding types: a) scavenging and predatory prey, b) suspension and deposit feeding prey, and c) non-determined feeding types. The characterization was based on the traits database biotic (www.marlin.ac.uk/biotic) and expert judgment by the authors. Many prey species exhibited both suspension and deposit feeding, as was the case for bivalves and brittlestar species that dominated the stomach contents of plaice.

### Data Accessibility

Fish abundance and infauna data for the Kattegat is already available through the Dyrad data repository (http://dx.doi.org/10.5061/dryad.04dv4). Stomach contents data for dab and plaice are available through DAPSTOM database held by CEFAS. Stable isotope as well as data on infauna, and fish from the Irish Sea is available through the Dyrad data respository (doi:10.5061/dryad.g3qh8).

## Electronic supplementary material

Supplementary information

## References

[1] Oberle FKJ, Storlazzi CD, Hanebuth TJJ (2016). What a drag: Quantifying the global impact of chronic bottom trawling on continental shelf sediment. J. Mar. Syst..

[2] Jennings S, Kaiser MJ (1998). The Effects of Fishing on Marine Ecosystems. Adv. Mar. Biol..

[3] van Denderen PD, van Kooten T, Rijnsdorp AD (2013). When does fishing lead to more fish? Community consequences of bottom trawl fisheries in demersal food webs. Proc. R. Soc. B.

[4] Link JSKB (2002). and C. G. M. The feeding ecology of flatfish in the Northwest Atlantic. J. Northw. Atl. Fish. Sci.

[5] Johnson AF, Gorelli G, Jenkins SR, Hiddink JG, Hinz H (2014). Effects of bottom trawling on fish foraging and feeding. Proc. R. Soc. B.

[6] Hiddink JG, Johnson AF, Kingham R, Hinz H (2011). Could our fisheries be more productive? Indirect negative effects of bottom trawl fisheries on fish condition. J. Appl. Ecol..

[7] Smith B, Collie J, Lengyel N (2013). Effects of chronic bottom fishing on the benthic epifauna and diets of demersal fishes on northern Georges Bank. Mar. Ecol. Prog. Ser..

[8] Choi JS, Frank KT, Leggett WC, Drinkwater K (2004). Transition to an alternate state in a continental shelf ecosystem. Can. J. Fish. Aquat. Sci..

[9] Shephard S, Brophy D, Reid DG (2010). Can bottom trawling indirectly diminish carrying capacity in a marine ecosystem?. Mar. Biol..

[10] Groenewold S, Fonds M (2000). Effects on benthic scavengers of discards and damaged benthos produced by the beam-trawl fishery in the southern North Sea. ICES J. Mar. Sci..

[11] Kaiser MJ, Ramsay K (1997). Opportunistic feeding by dabs within areas of trawl disturbance: Possible implications for increased survival. Mar. Ecol. Prog. Ser..

[12] Hill B, Wassenberg T (2000). The probable fate of discards from prawn trawlers fishing near coral reefs: A study in the northern Great Barrier Reef, Australia. Fish. Res..

[13] Bozzano A, Sardà F (2002). Fishery discard consumption rate and scavenging activity in the northwestern Mediterranean Sea. ICES J. Mar. Sci..

[14] Kaiser MJ, Spencer BE (1994). Fish scavenging behaviour in recently trawled areas. Mar. Ecol. Prog. Ser..

[15] Pinnegar JK, Polunin NVC (1999). Differential fractionation of *δ*^13^ C and *δ*^15^N among fish tissues: implications for the study of trophic interactions. Funct. Ecol..

[16] Michener, R. H. & Kaufmann, L. In *Stable Isotopes in Ecology and Environmental Science* 238–282 (2007).

[17] Caut S, Angulo E, Courchamp F (2009). Variation in discrimination factors (Δ^15^N and Δ^13^C): the effect of diet isotopic values and applications for diet reconstruction. J. Appl. Ecol..

[18] Perkins MJ (2014). Application of Nitrogen and Carbon Stable Isotopes (d^15^N and d^13^C) to Quantify Food Chain Length and Trophic Structure. PLoS One.

[19] Vanderklift MA, Ponsard S (2003). Sources of variation in consumer-diet d^15^N enrichment: a meta-analysis. Oecologia.

[20] Vander Zanden MJ, Cabana G, Rasmussen JB (1997). Comparing trophic position of freshwater fish calculated using stable nitrogen isotope ratios (δ 15 N) and literature dietary data. Can. Jouirnal Fish. Aquat. Sci..

[21] Owens NJP (1988). Natural Variations in ^15^N in the Marine Environment. Adv. Mar. Biol..

[22] Harrigan P, Zieman JC, Macko SA (1989). The Base of Nutritional Support for the Gray Snapper (Lutjanus-Griseus) - an Evaluation Based on a Combined Stomach Content and Stable Isotope Analysis. Bull. Mar. Sci..

[23] Rau GH, Takahashi T, Desmarais DJ, Repeta DJ, Martin JH (1992). The Relationship between Delta-C-13 of Organic-Matter and [Co_2_(Aq)] in Ocean Surface-Water - Data from a Jgofs Site in the Northeast Atlantic-Ocean and a Model. Geochim. Cosmochim. Acta.

[24] Fry B, Scalan RS, Parker PL (1977). Stable carbon isotope evidence for two sources of organic matter in coastal sediments: seagrasses and plankton. Geochim. Cosmochim. Acta.

[25] Hiddink JG (2016). Bottom trawling affects fish condition through changes in the ratio of prey availability to density of competitors. J. Anim. Ecol..

[26] Tableau AHLB, Brind’Amour A (2015). Available Benthic Energy Coefficient (ABEC): a generic tool to estimate the food profitability in coastal fish nurseries. Mar. Ecol. Prog. Ser..

[27] Loo L, Baden SP, Ulmestrand M (1993). Suspension feeding in adult *Nephrops norvegigus* (L.) and *Homarus Gammarus* (L.) (Decapoda). Netherlands J. Sea Res..

[28] Le Loc’h F, Hily C (2005). Stable carbon and nitrogen isotope analysis of *Nephrops norvegicus/Merluccius merluccius* fishing grounds in the Bay of Biscay (Northeast Atlantic). Can. J. Fish. Aquat. Sci..

[29] Hinz H, Kröncke I, Ehrich S (2005). The feeding strategy of dab *Limanda limanda* in the southern North Sea: Linking stomach contents to prey availability in the environment. J. Fish Biol..

[30] Stratoudakis Y, Fryer RJ, Cook RM, Pierce GJ, Coull Ka (2001). Fish bycatch and discarding in. Environment.

[31] Shephard S (2014). Scavenging on trawled seabeds can modify trophic size structure of bottom-dwelling fish. ICES J. Mar. Sci..

[32] Badalamenti F, Anna GD, Pinnegar JK, Polunin NVC (2002). Size-related trophodynamic changes in three target fish species recovering from intensive trawling. Mar. Biol..

[33] Badalamenti F (2008). Limited trophodynamics effects of trawling on three Mediterranean fishes. Mar. Biol..

[34] Bugoni L, Mcgill RAR, Furness RW (2010). The importance of pelagic longline fi shery discards for a seabird community determined through stable isotope analysis. J. Exp. Mar. Bio. Ecol..

[35] Ziegler, F. & Valentinsson, D. Environmental life cycle assessment of Norway lobster (*Nephrops norvegicu*s) caught along the Swedish west coast by creels and conventional trawls — LCA methodology with case study. *Int. J. LCA.* **13**, 487–497 (2008).

[36] Hinz H, Prieto V, Kaiser MJ (2009). Trawl disturbance on benthic communities: Chronic effects and experimental predictions. Ecol. Appl..

[37] Rijnsdorp AD, Vingerhoed B (2001). Feeding of plaice *Pleuronectes platessa* L. and sole *Solea solea* (L.) in relation to the effects of bottom trawling. J. Sea Res..

[38] Duineveld GCA, Van Noort GJ (1986). Observations on the population dynamics of *Amphiura filiformis* (ophiuroidea: echinodermata) in the southern North Sea and its exploitation by the dab, *Limanda limanda*. Netherlands J. Sea Res..

[39] Braber L, de Groot SJ (1973). The food of five flatfish species (Pleuronectiformes) in the southern north sea. Netherlands J. Sea Res..

[40] Cristo M (1998). Feeding ecology of *Nephrops norvegicus* (Decapoda: Nephropidae). J. Nat. Hist..

[41] Parslow-Willimas P, Goodheir C, Atkinson RJA, Taylor AC (2002). Feeding energetics of the Norway lobster, *Nephrops norvegicus* in the Firth of Clyde, Scotland. Ophelia.

[42] Kaehler S, Pakhomov EA (2001). Effects of storage and preservation on the *δ*^13^C and *δ*^15^N signatures of selected marine organisms. Mar. Ecol. Prog. Ser..

[43] Wood S (2015). Package ‘mgcv’. Mixed GAM Computation Vehicle with GCV/AIC/REML Smoothness Estimation. Version.

[44] Zuur, A. F., Ieno, E. N., Walker, N. J., Saveliev, A. A. & Smith, G. M. *Mixed effects models and extensions in ecology with R*. (Springer Verlag, 2009).

[45] Barnes C, Jennings S, Barry JT (2009). Environmental correlates of large-scale spatial variation in the d 13 C of marine animals. Estuar. Coast. Shelf Sci..

[46] Jennings, S. & Warr, K. J. Environmental correlates of large-scale spatial variation in the δ^15^N of marine animals, *Mar. Biol.* *142*, 1131–1140 (2003).

[47] MacKenzie KM, Longmore C, Preece C, Lucas CH, Trueman CN (2014). Testing the long-term stability of marine isoscapes in shelf seas using jellyfish tissues. Biogeochemistry.

[48] Post DM (2007). Getting to the fat of the matter: models, methods and assumptions for dealing with lipids in stable isotope analyses. Oecologia.

[49] Stephenson RL, Tan FC, Mann KH (1986). Use of stable carbon isotope ratios to compare plant material and potential consumers in a segrass and a kelp bed in Nova Scotia, Canada. Mar. Ecol. Prog. Ser..

[50] Rossi F, Herman PMJ, Middelburg JJ (2004). Interspecific and intraspecific variation of d13C and d15N in deposit- and suspension-feeding bivalves (*Macoma balthica* and *Cerastoderma edule*): Evidence of ontogenetic changes in feeding mode of Macoma balthica. Limnol. Oceanogr..

