# Supplementary material for: Stable isotopes reveal the effect of trawl fisheries on the diet of commercially exploited species
Source: Sci Rep. 2017 Jul 24;7:6334. doi: 10.1038/s41598-017-06379-6 (PMC5524968; doi:10.1038/s41598-017-06379-6)
Supplement: Supplementary file 1 — Supplementary information [file 41598_2017_6379_MOESM1_ESM.pdf]

Stable isotopes reveal the effect of trawl fisheries on the diet of commercially exploited species

Hilmar Hinz*[1], Joan Moranta[2+], Stephen Balestrini[3+], Marija Sciberras[3+], Julia R Pantin[3+], James Monnington[3+], Alex Zalewski[3+], Michel J Kaiser[3+], Mattias Sköld[4+], Patrik Jonsson[4+], Francois Bastardie[5+], Jan Geert Hiddink[3+]

Appendix

Table S1. Site characteristics of the sampling stations in the Irish Sea.

Study area	Times trawled (y-1)	Depth (m)	Shear stress (N m-2)	Annual Mean Bottom Temperature (Celsius)	MPD (mm)	Silt-clay (%)	Organic content (%)
B	7.3	31	0.22	10.74	0.083	58	5.1
C	5.8	26	0.24	10.70	0.088	52	5.3
D	8.8	42	0.22	10.81	0.088	52	0.7
E	10.5	38	0.21	10.78	0.074	76	6.4
G	9.4	29	0.21	10.72	0.075	71	0.8
I	7.8	38	0.19	10.82	0.091	49	4.4
L	7.7	36	0.19	10.82	0.079	63	5.4
O	11.9	31	0.18	10.79	0.070	85	6.4
R	3.6	28	0.18	10.83	0.074	74	5.8
S	6	24	0.23	10.83	0.074	74	6.0
T	2.7	24	0.28	10.83	0.075	71	5.9

Table S2. Site characteristics of the sampling stations in the Kattegat. Stations in italics and bold were excluded from further analysis because they combined higher fishing effort with coarser sediment and could therefore have confounded the outcomes.

Station	Depth (m)	Annual mean Temper-ature (Celsius)	Mud and silt (%)	Sediment 10% quantile (µm)	Sediment Median grain size (µm)	Sediment 90% quantile (µm)	Closure	Swept area ratio (y^{-1})
A	31	8.12	100	2	8	35	No take zone	0.4
B	27	8.38	100	3	9	36	No take zone	1.5
C	32	8.18	85	3	12	172	No take zone	0.8
D	31	8.23	99	3	10	60	No take zone	1.5
E	27	8.24	100	2	10	51	No take zone	0.2
F	28	8.43	99	3	20	76	Selective seasonal closure	2.6
G	31	8.53	100	2	9	29	Selective seasonal closure	4.6
H	34	8.27	100	3	10	38	Selective seasonal closure	4.6
I	34	8.50	100	3	9	32	Selective seasonal closure	4.5
J	31	8.37	78	3	13	223	Seasonal closure	7.9
K	***34***	**9.06**	***52***	***4***	***117***	***355***	***Seasonal closure***	***5.4***
L	34	8.41	100	3	10	44	Selective seasonal closure	5.7
M	33	8.27	100	2	8	30	No take zone	0.4
N	39	8.30	93	3	10	104	Selective seasonal closure	4.5
O	***32***	**8.60**	***43***	***4***	***156***	***344***	***Seasonal closure***	***8.0***
P	35	8.49	100	2	9	29	Selective seasonal closure	5.4
Q	***37***	**8.73**	***40***	***5***	***186***	***473***	***Open to all fishing***	***9.1***
R	38	9.16	94	3	21	107	Open to all fishing	7.4
S	***40***	**9.39**	***76***	***4***	***59***	***223***	***Open to all fishing***	***8.6***

Table S3. Relationships between predicted isotopic baselines ($\delta^{13}C_{base}$ and $\delta^{14}N_{base}$) and observed values for species in respective areas. Isotopic baselines were predicted from published regression models using sea bottom temperature to predict isotopic signatures of organisms at the base of the food web (here scallops). There were no correlations between the predicted isotopic baseline ($\delta^{13}C_{base}$ and $\delta^{14}N_{base}$) and the observed isotopic values measured in respective study areas suggesting that measured trends were unlikely to be caused by underlying baseline changes.

**Area**	**Variables**		**Intercept**	**Slope**	**r^2**	**p-value**
**Kattegat**	Predicted $\delta^{13}C_{base}$	Plaice $\delta^{13}C$	-16.696	0.050	0.010	0.757
		Nephrops $\delta^{13}C$	-20.454	-0.146	0.108	0.297
		Dab $\delta^{13}C$	-48.034	-1.544	0.075	0.343
	Predicted $\delta^{14}N_{base}$	Plaice $\delta^{15}N$	2.222	0.325	0.197	0.149
		Nephrops $\delta^{15}N$	9.181	-0.193	0.062	0.436
		Dab $\delta^{15}N$	20.328	-0.917	0.028	0.567
**Irish Sea**	Predicted $\delta^{13}C_{base}$	*Nephrops* $\delta^{13}C$	-17.134	-0.015	0.040	0.604
	Predicted $\delta^{14}N_{base}$	*Nephrops* $\delta^{15}N$	7.743	0.017	0.012	0.780

Table S4. To ensure that isotopic baselines variations did not drive or influence the relationships observed between isotopic values and trawling the GAMM analyses were repeated on the observed data subtracting predicted isotopic baseline data, i.e. the new response variable was the difference between observe isotopic values – the predicted baseline. The shape of the relationships (see S5) as well as the significance levels followed the trends observed in the original analysis (S4) demonstrating that baseline variation over the area had little influence on the observed relationships reported due to trawling. Isotopic baselines for both $\delta^{13}C$ and $\delta^{14}N$ were predicted from published regression models using sea bottom temperature to estimate isotopic signatures of organisms at the base of the food web (i.e. scallops[35-37] see methods). Extracted mean annual temperatures can be found in table S1 and table S2. Predicted mean baselines for the Kattegat a) $\delta^{13}C$ -17.62 ±0.11 S.D and δ14N 6.61 ±0.21 S.D. b) for the Irish sea $\delta^{13}C$=-16.87 ± 0.01 S.D. and $\delta^{14}N$ ±0.02 S.D. GAMM statistical outputs. Res.df are the residual degrees of freedom.

	N	Groups	Res.df	F	R^2	p	
**Kattegat**							
Observed Plaice $\delta^{13}C$ - $\delta^{13}C_{baseline}$	76	13	2.1	3.32	0.195	0.036	*
Observed Plaice $\delta^{15}N$- $\delta^{15}N_{baseline}$	76	13	2.95	2.95	0.045	0.089	
Observed Dab $\delta^{13}C$ - $\delta^{13}C_{baseline}$	85	15	2.06	2.62	0.124	0.093	
Observed Dab $\delta^{15}N$ - $\delta^{15}N_{baseline}$	85	15	1	2.82	0.074	0.096	
Observed *Nephrops* $\delta^{13}C$ - $\delta^{13}C_{baseline}$	68	12	1	7.21	0.151	<0.009	***
Observed *Nephrops* $\delta^{15}N$ - $\delta^{15}N_{baseline}$	68	12	2.54	7.17	0.227	<0.001	****
**Irish Sea**							
Observed *Nephrops* $\delta^{13}C$ - $\delta^{13}C_{baseline}$	52	9	1	6.40	0.172	0.014	**
Observed *Nephrops* $\delta^{15}N$ - $\delta^{15}N_{baseline}$	52	9	1.874	5.45	0.139	0.025	*

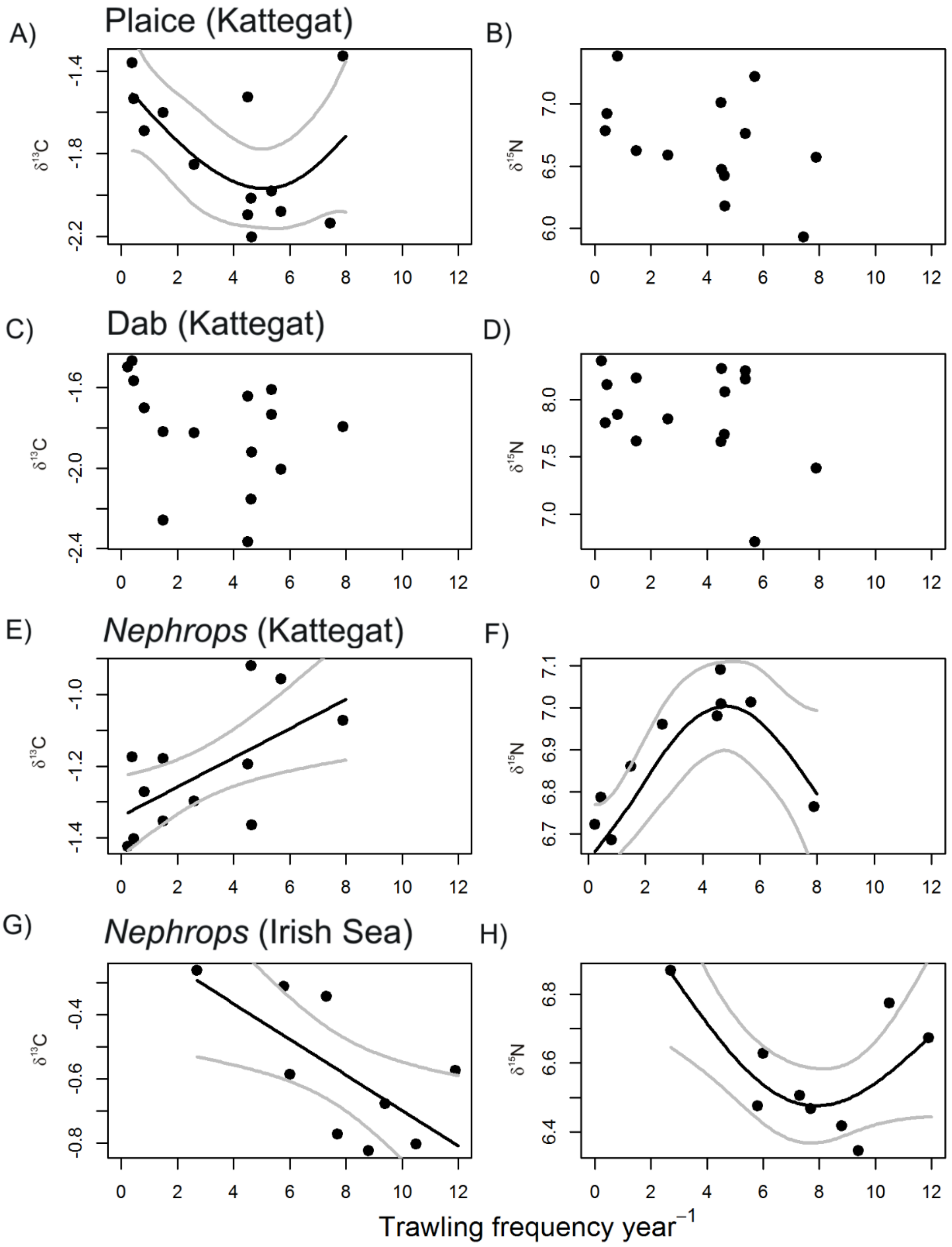

Figure S5. Relationships between the difference of observed $\delta^{13}C$ and $\delta^{14}N$ values and the predicted isotopic baselines $\delta^{13}C_{baseline}$ and $\delta^{14}N_{baseline}$ respectively (i.e. Observed – predicted baseline values) with trawling intensity. Summary of results can be found in table S3.
